# *Eurosurveillance* reviewers in 2025

**DOI:** 10.2807/1560-7917.ES.2026.31.1.2601081

**Published:** 2026-01-08

**Authors:** Eurosurveillance editorial team

**Affiliations:** 1 European Centre for Disease Prevention and Control (ECDC), Stockholm, Sweden

**Keywords:** peer-review

The editorial team is grateful to all the experts who dedicated their time to peer-review our articles over the past 12 months. We wish to thank them and all those whose names are not listed below but gave helpful advice and support. We apologise to any reviewers whose names we may have missed.

Our reviewers in 2025 were:

Jaime David Acosta-España; Paul Adamson; Suman Adhikari; Anton Aebischer; Seven Johannes Sam Aghdassi; Antonella Agodi; Harit Agroia; Philipp Agyeman; Alexandre Alanio; Steffen Albrecht; Haider Al-Hello; Shohaib Ali; Lao-Tzu Allan-Blitz; Vanessa Allen; Erik Alm; Claudia Alteri; Maria Joao Alves; Rishma Amarsy; Antonella Amendola; Fabrizio Anniballi; Spinello Antinori; Patricia Antunes; Takeshi Arashiro; Carmen Ardanuy; Barry Atkinson; Hiroaki Baba; Laura H. Bachmann; Heather Bailey; Jean-Luc Bailly; Tamas Bakonyi; Mathieu Bangert; Cyril Barbezange; Francesco Barchiesi; Silvia Barros; Luisa Barzon; Nathalie Bastien; Ulrike Baum; Frank Beard; Julien Beauté; Kimberley Benschop; Bojana Beovic; Beatrice Bercot; Maël Bessaud; Boris J B Beudeker; Sangeeta Bhatia; Adarsh Bhimraj; Melanie Bissessor; Julia Bitzegeio; Jeppe Boel; Pierre Bogaerts; Chara Bogogiannidou; Otilia Boldea; Shubhada Bopegamage; Valerio Bordino; Eva Borràs; Jordi Borrell Pique; Ray Borrow; Alessio Bortolami; Emmanuel Bottieau; Anders Boyd; Francesco Branda; Arne Brantsæter; Carina Brehony; Eeva Broberg; Colin Brown; Lynda Browning; Andrea Büchler; Hanna Buck; Niccolò Buetti; Irene Burckhardt; Alba Cabré-Riera; Daniel Cadar; Jeremy Camp; Rosa Cano; Christina Carlander; Heather Carleton; David Carmena; Jordi Casabona; Jean-Sebastien Casalegno; Marta Castelhano; Jesus Castilla; Gaud Catho; Vincent Cattoir; Simon Cauchemez; Orlando Cenciarelli; Marianne Chemaly; Yaolong Chen; Alexandra Chiaverini; Lydia Chitimia-Dobler; Olivier Chosidow; Gerardo Chowell; Iva Christova; Jessie Chung; Jose Miguel Cisneros; Heike Claus; Hazel Clothier; Anne Conrad; Martin Cormican; Laura Cornelissen; Dominique Costagliola; Benjamin Cowling; Joseph Cox; Pierre Alex Crisinel; Tine Dalby; Fortunato D'Ancona; Eric Dannaoui; Raymund Dantes; Stefan Dascalu; Ed Davila; Kimberly Davis; Brechje de Gier; Daniel de Vries; Martin Dedicoat; Jean-Claude Desenclos; Phillippe Dewals; Antonino Di Caro; Asuncion Diaz; Brent Dixon; Katarzyna Domańska-Blicharz; Amy Douglas; Aparna Dressler; Jan Drexler; Rikard Dryselius; Erika Duffell; Robert Dyrdak; Michael Edelstein; Armin Elbers; Hanne-Dorthe Emborg; Hélène Englund; Olivier Epaulard; Conrada Erkens; Sylvie Escolano; Laura Espinosa; Vicente Estrada; Massimiliano Fabricci; Gerhard Falkenhorst; Dennis Falzon; Daniele Fasan; Nicolás Francisco Fernández Martínez; Patricia Pita Ferreira; Julie Figoni; Antonietta Filia; David Fisman; Stefan Flasche; Antje Flieger; Anne Fouillet; Christina Frank; Neil Franklin; Maria Fredriksson-Ahomaa; Atle Fretheim; Caroline Frey; Ana Friães; Hagen Frickmann; Ingrid Friesema; Niels Frimodt-Møller; Silvia Funke; Giovanni Gabutti; Wolfgang Gaissmaier; José-María García-Carrasco; Patricia Garvey; Silvana Gastaldi; Tatjana Gazibara; Nancy Gerloff; Antoine Gessain; Marta Giovanetti; Aharona Glatman-Freedman; Federico Gobbi; Matthieu Godinot; Richard V. Goering; Fernando Gonzalez-Candelas; Aikaterini Grimani; Tamar Grossman; Beate Grüner; Larisa Gubareva; Mariana Guedes; Rasmus Gustafsson; Jean-Paul Guthmann; Christos Hadjichristodoulou; Ferry Hagen; Susan Hahne; Ágnes Hajdu; Luisa Hallmaier Wacker; Anette Hammerum; Brett Hanscom; Lisa Hansen; Thomas Harder; Ross Harris; Heli Harvala; Heli Harvala; Richard Hassall; Anja Hauri; David TS Hayman; Qiushui He; Wen-Qiang HE; Jelte Helfferich; Antoni Hendrickx; Niel Hens; Victoria Hernando; Laura Herrera-León; Christian Herzog; Maya Holding; Franziska Hommes; Vivian Hope; James Humphreys; Susanne Hyllestad; Heikki Ilmavirta; Victoria Indenbaum; Alexander Indra; Raihana Jabbar; Manon Jaboyedoff; Stéphanie Jacquinet; Niklas Jakobsson; Klaus Jansen; Benoit Jaulhac; Cecilia Jernberg; Nicholas Johnson; Pikka Jokelainen; Kai Jonas; Gabrielle Jones; Vita Jongen; Sandra Junglen; Annette Jurke; Tsutomu Kageyama; Bernard Kaic; Suzanne Kalb; Hajime Kanamori; Tommi Karki; Ariel Karlinsky; Johan Karp; Ludmila Kasatkina; James D Kellner; Harald Kessler; Nino Khetsuriani; Amir Kirolos; Peter Kirwan; Esther Kissling; Ian Kitai; Charlotte Kjelsø; Lars Kåre Selland Kleppe; Michael Klompas; Nobumichi Kobayashi; Anke Kohlenberg; Katarina Kowatsch; Sarah Kramer; Tyra Krause; Manuel Krone; Catarina Krug; Marcela Krutova; Sharon Kühlmann-Berenzon; Takako Kurata; Sanja Kurečić Filipović; Angie Lackenby; Heimo Lagler; Katrien Lagrou; Margaret Lam; Theresa Lamagni; Francois-Xavier Lamy; Phung Lang; Joanne Langley; Regina Larocque; Judith Leblanc; Jean-Daniel Lelièvre; Stephen Liang; Michael Libman; Ryanne W. Lieshout-Krikke; Giedrius Likatavicius; Monika Liptakova; Samuel Lipworth; Carmen Lisboa; Bette Liu; Emma Löf; Frederikke Lomholt; Ira Longini; Guillem López de Egea; Noemí López-Perea; Åke Lundkvist; Michael Lydeamore; Victoria D Lynch; Outi Lyytikäinen; Noni MacDonald; Shingai Machingaidze; Fabrizio Maggi; Fabio Magurano; Alexandra Mailles; Maria Francesca Manca; Alessandro Mancon; Michal Mandelboim; Sierk D Marbus; Otilia Mårdh; Michael Marks; Robby Markwart; Victoria Marriott; Gaetano Marrone; Jonas Marschall; Katherine E. Marshall; Esteban Martinez; Isabel Martínez Pino; Josefa Masa-Calles; Wesley Mattheus; Fiona May; Lesley McGee; Anna McNaughton; Adam Meijer; Eelco Meijer; Ella Mendelson; Silja Mentula; Dick Menzies; Yanis Merad; Christina Merakou; Eric Meyerowitz; Sofie Midgley; Kei Mikita; Massimo Mirandola; Ali Mirazimi; Reiko Miyahara; Geert Molenberghs; Thomas Mollet; Susana Monge; Arnold Monto; Marco Moretti; Marina Morgan; Vanessa Morton; Davide Moschese; Joël Mossong; Laurent Moulin; Antons Mozalevskis; Judith Mueller; Miguel Mulders; Vincent Munster; David Muscatello; Aniket Nadkarni; Celine Nadon; Anita Nagy; Neda Nasheri; Tony Nelson; Nathalie Nicolay; Christoph Niederhauser; Stine Nielsen; Andreas Nitsche; Francisco Nogareda; Hanna Nohynek; Anne Christine Nordholm; Lucia Nova; Norbert Nowotny; Olisaeloka Nsonwu; Nicholas Ogden; Piaras O'Lorcain; Richard Osei-Yeboah; Joshua Osowicki; Bas Oude Munnink; Romain Palich; Vassiliki Papaevangelou; Katherine Paphitis; John Papp; Georgios Pappas; Maria Pardos de la Gandara; Elena Pariani; Ben Pascoe; Lucia Pastore Celentano; Dipti Patel; Lara Payne; Junping Peng; Justin Penner; Eskild Petersen; Tamas Petrovic; Patrizio Pezzotti; Martin Pfeffer; Yvonne Pfeifer; Sarah E. Philo; Denis Piérard; Johann David Daniel Pitout; Andreas Plate; Catherine Plüss-Suard; Anne Pohlmann; Piero Poletti; Bastian Prasse; Katarina Prosenc; Caroline Quach; Thai Quan; Hardis Rabe; Angelo Roberto Raccagni; Jose M Ramos; Moa Rehn; Jacqui Reilly; Juliana Reyes-Urueña; Flavia Riccardo; Enrico Ricchizzi; Maria Luisa Ricci; Wendy Rice; Thomas V. Riley; Alberto Rizzo; Annapaola Rizzoli; Lucy Robertson; Emmanuel Robesyn; Miguel Rocha; Rafael Rocha; Rachel Roche; Anne-Marie Roque-Afonso; Senia Rosales-Klintz; Angela Rose; Bettina Rosner; Francois Roubille; Jamie Thomas Rudman; Wallis Rudnick; Werner Ruppitsch; Leila C Sahni; Raquel Sá-Leão; Saara Salmenlinna; Gina Samaan; Frank Sandmann; Sabine Santibanez; Paul Saunderson; Vered Schechner; Simone Scheithauer; Patricia Schlagenhauf; Martin Schlaud; Jonas Schmidt-Chanasit; Eli Schwartz; Birgitta Schweickert; Joana Sequeira Neto; Jeong Hwan Shin; Carlo Signorelli; Lotta Siira; Reina Sikkema; Ian Simms; Ritu R Singh; Wouter L Smit; Rami Sommerstein; Klara Sondén; Michaela Špačková; Gianfranco Spiteri; Hein Sprong; Aurora Stanescu; Melanie Stecher; Gyde Steffen; J.A. (Arjan) Stegeman; Dominik Stelzle; Franc Strle; Michael Stucki; Lorenzo Subissi; Carl Suetens; Hideyuki Takahashi; Emi Takashita; Emilie Talagrand-Reboul; Gillian Tarr; Beatrice Tassis; Buhari Teker; Bernd-Alois Tenhagen; Heidi Theeten; Robyn Thorington; Alma Tostmann; Alberto Tozzi; Raymond Tsang; Ashleigh Tuite; Mugen Ujiie; Soren Uldum; Tim Uyeki; Francesco Vairo; Palle Valentiner-Branth; Dominique Van Beckhoven; Birgit van Benthem; Wim Van Bortel; Nicoline van der Maas; Sylvie van der Werf; Suzanne van der Werff; Steven van Lelyveld; Maaike van Mourik; Annelies Van Rie; Dominique Vandekerchove; Florent Vanstapel; Carmen Varela Santos; Tuula Vasankari; Julio A. Vázquez Moreno; Tomas Vega-Alonso; Guillaume Velut; Giulietta Venturi; Anne Vergison; Lasse Vestergaard; Marco Vinceti; Maartje Visser; Ioanna Voulgaridi; Emilia Vynnycky; Jiří Wallenfels; Teresa Walz; Haoyi Wang; Michael Ward; Amelia Warren; Adilia Warris; Merav Weil; Dirk Werber; Lukas Weseslindtner; Darren Westphal; Heather Whitaker; Ursula Wiedermann; Hendrik Wilking; Inge Willemstein; Sarah Williams; Maike Winters; Guanghui Wu; Barbara Yawn; Nicolas Yin; Hans L Zaaijer; Philipp Zanger; Peter Zarb; Jean-Pierre Zellweger; Lei Zhou; Ran Zhou; Neta Zuckerman; Anne Østergaard.

